# "The Hospital's" Commission on Light Wines

**Published:** 1907-06-15

**Authors:** 


### "THE HOSPITAL'S" COMMISSION ON LIGHT WINES.

### A SPECIAL REPORT

ON

### THEIR CHEMICAL COMPOSITION AND DIETETIC VALUE.

In a former report in these columns we dealt with the manufacture and properties of whisky; in the present article we intend to direct our attention to wines. Although wines are by the public —and, indeed, by the Inland Revenue authorities —looked upon as differing mainly with regard to alcoholic strength, even the cursory reader of what follows will soon gather that one wine may differ from another almost as much as a wine differs from a spirit. If wines can differ to such an extent chemically, it is not surprising that they affect their consumers in such various ways.

In order that our readers, whom we assume to be mainly medical practitioners, shall not be disappointed, we will at once confess the weakness of our method and the indefiniteness of the conclusions which we have been able to draw from our experiments. In the present state of physiological and pharmacological knowledge, it is impossible from mere chemical data to predict what wine will be harmful and what beneficial to a given patient. If we enlarge the scope of our experimental field and include in it, as we have done, observations upon the effects of adequate quantities of different wines upon the actual chemical processes of digestion, we obtain data from which more particular inferences can be drawn; but even after an elaborate series of such experiments the conclusions will be but general, and from them we shall be able to indicate, not what individual wine, but only what class of wine, would be likely to suit any given class of patient.

### The By-Products.

In discussing the physiological action of spirits, or rather of whisky, we pointed out that the effect of the whole was due to the sum of the effects of the parts. The same dictum is to all intents and purposes true of wines. The common factor in wines and spirits is alcohol, and, as with brandy and whisky, so in the case of wines, the effects are due to the alcohol *plus* individual by-products. The by-products of wine are of an absolutely different chemical class to the by-products of spirits, and with, perhaps, the small exception of brandy, which is the most vinous spirit we possess, there is no physiological kinship between these two classes of beverages. In other words, the higher alcohols, compound ethers, aldehydes, and empyreumatic bodies in spirits may conveniently be regarded as homologous to the extract, acid, and sugar of wines, but are in no sense analogous to them. We shall have subsequently to enter at some length into the action of what we have, for the sake of lucidity, termed the by-products of wines, but before doing this we must fulfil the invidious function of quarrelling with our own terminology. We have referred to the extract as being a by-product. If by the term "extract" we mean the non-alcoholic residue of wines, we must at once emphasise the fact that this constituent, or rather these constituents, of wine bear quantitatively quite a different proportion to the alcohol than is the case in spirits. For instance, in the case of a well-known claret (Larose) the alcohol is 10.01 per cent. (by volume), whereas the residue amounts to 2.4 per cent., or about one-quarter of the alcohol. In the case of an old Irish whisky, with an alcoholic content of 50 per cent., the total by-products, including under this term the higher alcohols, would amount to less than 0.3 per cent., or one two-hundredth of the alcohol.

### The Temperance Problem.

Those of us who have read the immortal Boswell will remember the praise bestowed by Dr. Johnson upon the wine of a host who was ambitious to please the palate of the philosopher. "The wine is red, thick, and it makes you drunk. What more do you want?" said Dr. Johnson. The last, and indeed the least, important function of wine, or good wine, is that it intoxicates, and hence the properties of wine were put in the right order by the sage. *It cannot too emphatically be insisted upon that light wine is essentially a temperance beverage, and that its alcoholic content is in many cases a character of secondary importance.* Without alcohol one cannot have wine, because it is only the alcoholic fermentation of the sugars produced in the must or raw grape-juice by certain yeasts that will give us the products which we prize and which constitute the extract and the bouquet.*

Wine, in the language of the Psalmist, is to make glad the heart of man, and is not to intoxicate him. One can only get sufficient alcohol from wine to produce its full action of making one drunk by

* The composition of wine varies according to the grapes and according to the year. The sugars of the must consist of almost equal parts of dextrose and lævulose, the latter being usually slightly in excess.

taking sufficient extract to make one ill; wine is not the drink of the dipsomaniac, but of the *viveur*.

From these remarks it must not be inferred that wine has not been used, and will not be used, for the purpose of producing drunkenness, but we have sound reasons for presuming that the pathological effects of chronic alcoholism is much more likely to be produced from spirits than from wine. The abuse of wine produces other effects, not so much due to the alcohol as to the extract—namely, hepatic inefficiency, goutiness, and other phenomena dependent upon disorganisation of metabolism. Gout, plethora, and apoplexy were the punishments more commonly meted out to the winebibber of Georgian times, whereas cirrhosis, neuritis, and affections of the central nervous system—in short, purely alcoholic effects—are the usual penalties which the modern spirit drinker pays for his excess. The former are much easier to correct or mitigate than the latter, which are practically incurable. We get a very good insight into what we may term the wine intemperance of the regency and the subsequent reign of George IV. in the Creevey Memoirs. The admiration of Mr. Creevey for Sheridan, who was notoriously a wine drinker, is interesting in this connection. Sheridan had retired to rest apparently quite overcome by alcohol at about 11 p.m., but, to Mr. Creevey's great surprise, he reappeared again at a ball at about 2 a.m. quite sober, and enjoyed another bottle of wine. The spirit intemperate of to-day would find it exceedingly difficult to do this.

### The Physiological Action of Alcohol.

There is probably scarcely a subject upon which so much has been written as upon the physiological action of alcohol. From the actual experimental data available the extremists upon each side of the controversy have found to their own minds adequate material to support their individual views. In this article we are concerned with dietetics, and not with economics, and although we propose giving a brief account of the action of alcohol, as this substance forms an integral part of wine, we shall avoid controversial matters, although no one can refuse to admit that chronic intemperance is the most active cause of crime and disease at present extant. With regard to crime, the statistics of Baer are of especial interest. This observer, in an examination of no fewer than 32,837 prisoners, found 41.7 per cent. to be sufferers from chronic alcoholism. In proper doses taken at suitable times alcohol can be truly described as a friend to man; but in excess, and especially when associated, as so often happens among the poorer classes, with insufficient diet, it can be easily converted into his bitterest enemy.

For the sake of lucidity we shall consider the action of alcohol under the following headings:—

#### *The Fate of Alcohol in the Body.*

Upon this question hinge the very rudiments of the controversy as to whether alcohol is to be considered a drug or a food. By a food we mean a substance which is split up or decomposed in the body into simpler constituents, and, by virtue of this splitting up, supplies energy to the body. As a matter of fact, we know that the truth here lies between the two extreme opinions, and that, although alcohol is not fully oxidised in the body, yet it is very nearly so when taken in a moderate dose of 30 c.c., such as is contained in two ounces of whisky or eight ounces of wine. Of the small percentage of alcohol—at the most 10 per cent.—unchanged in the body, the major part leaves the body by the kidneys and the rest by the lungs.

#### *The Effect of Alcohol upon Digestion.*

Quantities of alcohol up to from 5 to 8 per cent. seem to have either a favourable or no effect upon the digestive processes. If we take the stomach content during a meal at approximately a quart, this means that during a meal about eight ounces of wine of average alcoholic strength can be consumed without exerting any injurious effect, *qua* alcohol, upon the chemical processes of digestion. In making this estimate we must remember that alcohol is absorbed very rapidly by the healthy mucous membrane, and that therefore if he takes care to sip his wine while eating food there is little fear of the moderate wine-drinker ever in the course of a meal obtaining in his stomach 5 per cent. of alcohol. In this connection we must observe that the absorption of wine by the mucous membrane of the stomach seems to stimulate the secretion of both the acid and the pepsin of the gastric juice. This physiological function of wine is similar to that of salted appetisers, as for instance caviare, and explains the use of a glass of wine taken half an hour or a quarter of an hour before a meal. Such use of wine is often most beneficial to patients of feeble digestion. A busy man is very apt to continue his business until the last moment, rush home by some rapid means of locomotion, change his clothes as quickly as he can, and at once begin his dinner. Such a person sits down to his meal with his blood everywhere but where it ought to be—namely, in his abdomen—and with the waste products evolved during his day's work stagnating in his blood and in the organs which have produced them, giving rise to a sense of fatigue. The ideal advice to such a person is to rest for half an hour before beginning his meal; but rest at the beginning of the twentieth century is an unwelcome prescription, and the doctor who enforces it is apt to share the fate of the hospitable innkeeper in "The Three Musketeers," and be obliged, like him, to take down his sign. Failing rest, a glass of good wine is the best remedy. Its bouquet stimulates reflexly through the palate the secretion of the digestive juices; its alcohol is quickly absorbed by the stomach, and this absorption directly influences the gastric mucous membrane, as we have mentioned. After absorption the alcohol and by-products accelerate the circulation, and thus hasten the excretion of the waste products of the day's work, and therefore remove fatigue.

#### *The Effect of Alcohol upon Respiration.*

According to the work of modern observers, a moderate dose of wine is a respiratory stimulant; that is, it increases the intake of oxygen and the output of carbon dioxide. We owe the most exact work upon this interesting subject to Zuntz and

Geppert,* who find that there is no difference between the action of wine upon the respiratory exchange and that of any other food. Their observations have been continued by Hewugean, according to whom eight ounces of wine of average alcoholic strength is equivalent to half a pound of bread.

### *Nature of the Food Value of Alcohol.*

The last point we shall discuss in this connection is the class of food to which wine essentially belongs. The relation of food of all kinds to the body may be roughly compared to that of the coal in the tender of a steam engine to the steam engine in motion. In the conversion of the latent chemical energy of coal into the kinetic energy of an express train some waste takes place. The coal has to convert water into steam, and the steam has to be made to expand in definite directions to produce the desired result. Furthermore, the ashes left behind by the combustion of the coal have to be removed. Every engineer knows that one coal differs from another in the amount of kinetic energy which can be got out of a given mass of it. So it is with food. Food must be digested before it can be assimilated, and the refuse of it removed; part of the energy derived from any given food is converted into that individual form of biological energy which is represented by the functions of digestion, absorption, and excretion. In this respect foods differ largely. Wine in moderate doses is a food from which there is practically no waste; it requires no digestion, and is rapidly absorbed from the stomach. Its food value as represented by the alcohol it contains is theoretically the greatest of any nutritive substance we possess, with the single exception of fat; and if we take into consideration the energy required to digest fat, the former is probably more economical. One gram of alcohol is capable of providing 7,184 calories, one gram of fat 9,300, one gram of proteid 5,711. Numerous experiments have been made as to the effect of given quantities of alcohol upon the amounts of other foods required to constitute a typical diet, and it has been clearly shown that alcohol is capable of actually replacing fat in the diet and of reducing the amount of proteid required. The most exhaustive experiments have been made by Neumann, † who found that if from the adequate diet of a working-man half the fat be removed, the tissue substance of the body is drawn upon to make up the deficiency, and the man accordingly wastes. If, however, the fat in question be replaced by wine in the proportion of approximately one gram of alcohol to two-thirds of a gram of fat, the diet remains adequate and the subject no longer wastes. Thus wine, by virtue of its alcohol content, is as much a food in the true sense of the word as fat. A further function of alcohol in this connection comes out of Neumann's experiments, and also from those of Minra and others, and is of especial importance with regard to wine—that this latter substance in small quantities has a favourable influence upon the actual absorption and use to the body of other foodstuffs, the body getting more energy from its proteids on a diet containing alcohol than upon one free from alcohol.

* Pfluger's "Archiv f. d. ges. Physiol." 1883, xxxii. 222.
† Archiv. f. Hygiene, 1899, xxxvi. 1.

### Alcohol a Food.

We find then that wine, simply regarding its alcoholic content, is capable of being oxidised by the body, of stimulating appetite, favouring the chemical processes of digestion, replacing so important a dietetic element as fat, and helping the body to make better use of another kind of food—namely, proteid. We venture to think, however, that in the literature of this subject too much has been made of the question of the actual food value of alcoholic beverages. Alcohol is a food, and it can be taken in doses in which it acts only as a food; but in practical dietetics the actual food value of a wine is of secondary importance. We do not regulate our meals according to the food value of their constituents; we want a pleasant meal and a pleasant drink, and the physiological value of pleasurable eating and pleasurable drinking is none the less real because we cannot measure it calorimetrically. The artful Jael beguiled the warrior Sisera not with butter only, but with butter in a lordly dish. At meals we have not only to do with the science of dietetics, but with the art of gastronomics. Wines may or may not add to the food value of a meal. Even if wine were not a food, in proper and suitable doses it is an accessory to food and a handmaiden to digestion. In the choice of wine for the stomach, as in the choice of grease for a machine, we must take care that we use it neither in quantity nor in quality to impair our machinery, and the object of the experiments which follow is to endeavour to decide what quantity and what quality of wine should be used.

### A Chemical Investigation of Light Wines.

The first division of our inquiry is essentially chemical, and this indeed in some respects is a most arduous one. The number of wines is legion, and their chemical compositon exceedingly variable. We have limited our survey to relatively light wines or to wines of low alcoholic content—in other words, to wines the vinous extract of which bears a considerable proportion to the alcohol. The wines which we have examined are French clarets, including sauterne and graves, moselles, hocks, champagnes, Australian clarets, and one sample each of Spanish and Italian clarets. Obviously, in considering the choice of a wine one cannot neglect the element of price, and in choosing the samples for experimental purposes we have endeavoured to include in each class of wine specimens of moderate cost as well as more expensive ones. In the following table, which gives the chemical composition of the wines in question, the first column gives the alcoholic content in volume per cent., the second, third, and fourth columns the extract, acid, and sugar respectively in grammes per litre. By the term "extract" of wine is understood the total quantity of non-volatile residue which is obtained by evaporating the wine to dryness. The extract constitutes what is known as the body of wine, and is the main chemical basis of the taste. Its constituents are for the most part unfermented sugar (including pentoses), nitrogenous compounds, non-volatile acids, tannin compounds, colouring matter, mineral substances, and nitrogen-free extractives. Wine also contains glycerine: the quantity of this substance in a genuine wine bears a proportion to the quantity of alcohol; according to Pasteur, for every 50 parts of alcohol there should be 3.5 parts of glycerine, but as a matter of fact the proportion varies within much wider limits. The acids of wine partly arise from those originally present in the must or raw grape juice, and partly as by-products of fermentation. They are chiefly malic and tartaric, with traces of succinic and acetic acids. Butyric acid is only present in wine which has undergone secondary or undesirable fermentation. Lactic acid in wine may also be the result of secondary fermentation, but in so far as some lactic acid is developed from the malic acid normally present, it must be regarded as a normal constituent of wine. An important constituent of wine from the medical standpoint is tannin. Most of the tannin present arises from the grape skins and pips; good white wine contains about 0.02 per cent. tannin, and if the percentage is over 0.05 per cent. an astringent taste is given to the wine. Red wine shows as a rule a percentage of tannin varying from 0.1 to 0.25 per cent.; here anything over 0.3 per cent. imparts an astringent taste to the wine. The colour of wine is due to two classes of compounds—chlorophyll and its derivatives, and the blue colouring matter of grapes. The bouquet and aroma-giving substances of wine arise from three sources. The first source is the grapes themselves; practically all grapes possess a special taste and smell which they impart to their respective wines. The second source is fermentation; different kinds of yeast seem to produce different kinds of aroma and bouquet-giving substances, and also different quantities of these. The last source of these important substances is the ripening and the maturing of wine. The substances produced in this way are for the most part the ethers corresponding to the volatile and the non-volatile acids present in the wine. We have entered, in some detail, into these various constituents of wine because it is not only upon the absolute quantity of them present in a given wine that an opinion with regard to the soundness or unsoundness of such a wine is based, but upon their proportion. In the table which follows we have not enumerated the individual constituents discussed above, with the exception of acid and sugar. These are from the medical standpoint important, and will be considered later.

Table I.—Showing the Chemical Composition of the Wines used in this Investigation.

N.B.—Alcohol in volume per cent., other constituents in grammes per litre.

| Wine | Alcohol | Extract | Acid | Sugar |
|---|---|---|---|---|
| *A. French Clarets.* | | | | |
| Ch. Margaux ... | 12·68 | 25·35 | 2·92 | — |
| Ch. Haute Lafite... | 12·48 | 27·10 | 3·28 | — |
| Bordeaux ... ... | 10·70 | 27·24 | 3·48 | — |
| Médoc ... | 10·25 | 23·39 | 3·16 | — |
| Ch. Margaux ... | 11·60 | 29·99 | 3·30 | — |
| Ch. Larose ... | 10·01 | 24·09 | 3·28 | 1·08 |
| Ch. Brane-Cantenac | 11·68 | 23·44 | 3·02 | — |
| Ch. d'Issan ... | 9·91 | 20·80 | 2·76 | 0·92 |
| Sweet Sauternes ... | 11·68 | 36·12 | 3·97 | 14·0 |
| Dry Sauternes ... | 12·78 | 29·87 | 4·06 | 3·0 |
| Haut Graves ... | 12·00 | 28·34 | 4·56 | 2·7 |
| Graves ... ... | 12·27 | 34·16 | 4·99 | 4·0 |
| *B. Moselles.* ... | | | | |
| Moselle (1) ... | 9·91 | 20·07 | 4·25 | — |
| Schwarzberger ... | 11·14 | 21·60 | 4·07 | — |
| Moselle A. ... | 10·66 | 20·26 | 4·23 | — |
| Moselle '93 ... | 11·41 | 22·28 | 4·51 | — |
| Moselle W. ... | 9·23 | 18·31 | 3·63 | 1·35 |
| Brauneberger ... | 11·67 | 20·99 | 3·63 | 1·51 |
| *C. Hocks.* | | | | |
| Hock 1 ... ... | 11·41 | 19·06 | 2·98 | — |
| „ 2 ... ... | 9·23 | 25·84 | 3·49 | — |
| „ 3 ... ... | 9·74 | 23·52 | 2·80 | — |
| „ 4 ... ... | 10·89 | 59·77 | 6·30 | 30·00 |
| „ 5 ... ... | 9·76 | 22·36 | 4·02 | 1·51 |
| *D. Champagnes.* | | | | |
| Champagne, 1 ... | 13·70 | 35·70 | 4·02 | 19·2 |
| „ 2 ... | 13·90 | 35·11 | 4·21 | 18·4 |
| „ 3 ... | 13·48 | 35·20 | 4·12 | 15·6 |
| „ 4 ... | 10·61 | 71·16 | 4·96 | 55·0 |
| *E. Australian.* | | | | |
| Australian, 1 ... | 14·20 | 29·65 | 2·96 | — |
| „ 2 ... | 16·19 | 36·68 | 2·98 | 2·84 |
| „ 3 ... | 13·39 | 29·15 | 4·05 | 1·08 |
| „ 4 ... | 13·16 | 25·04 | 3·30 | 0·92 |
| „ 5 ... | 14·48 | 37·40 | 4·17 | 2·57 |
| „ 6 ... | 13·25 | 28·46 | 3·97 | 1·63 |
| „ 7 ... | 15·46 | 88·27 | 4·21 | 50·00 |
| „ 8 ... | 15·36 | 30·24 | 4·02 | 2·57 |
| *F. Spanish.* 1 ... | 13·60 | 33·66 | 3·63 | 8·08 |
| *G. Italian.* 1 ... | 10·79 | 22·90 | 3·63 | 1·00 |

### The General Composition of Wines.

Before entering into the technique and results of our physiological experiments we think it advisable to offer a few remarks upon the general composition of wines.

### Clarets.

*Claret* may be divided roughly into two classes—namely, beverage and dessert wines. The difference between these two classes is merely one of degree, the former comprising the less, the latter the more expensive wines. The beverage wines are, as a rule, not classified according to vintage or the immediate vineyard from which they emanate, but are named according to the district in which they are grown. The main districts in which the Bordeaux clarets are grown are those of Médoc, Graves, and St. Emilion; these are further sub-divided, the Médoc, for instance, containing the sub-districts or communes of St. Julien, Pauillac, St. Estephe, and so on. A wine bearing any of these names—*i.e.*, the name of a district—would be regarded as a "beverage" as contrasted with a "dessert" wine, the latter adjective being applied to fine wines of specific vintages * and grown on certain specified properties, such as Château Margaux, Ch. Lafite, etc. There is a large intermediate class of wines which are "dessert" wines in the sense that they are classified according to vintage and vineyard (Château) and

* The "vintage" or year in which the claret was made is of great importance. In modern times the years 1875, 1888, 1896, and 1900 may be mentioned as among the most successful vintages recorded.

"beverage" wines in the sense that they are moderate in price. In regard to the considerations of chemical composition which connote the different shades of quality in French clarets, it is generally the case that fine wines contain a constant proportion of the various constituents; that is to say, the alcohol, extract, acid, and so on stand in certain proportion to each other. Compared with light red wines from other parts of the world, the French clarets contain a relatively low proportion of alcohol and extract, very little acid and tannin, and mere traces of sugar, and combine these chemical properties with a fine and delicate flavour. It is almost invariably the rule with wines of the claret type produced elsewhere than in the Gironde district that full flavour and bouquet are accompanied by high extract, and high acid and tannin values; generally also by the presence of sugar. In the Château wines of the Gironde (see Ch. Larose, Ch. Brane Cantenac, etc., in the table) we have light red wines of appetising flavour and taste, with a relatively low extract and low acid, and scarcely any sugar. Perhaps it may be said that in this connection we are going rather beyond our chemical figures. It must be admitted that in a sense this is so, but chemical figures unfortunately give us but little information with regard to taste and flavour. The substances imparting these all-important properties to wine are present in very minute quantities, and indeed up to the present have eluded chemical identification. Just as the human olfactory sense can detect the presence of musk in a quantity chemically undemonstrable, as too an unweighable quantity of abrin will cause the death of an animal, so also acute pleasure may come to the palate and consequent stimulation to the digestive processes from the taste of a fine wine, the chemical basis of the bouquet of which is quite unknown. All that we do know is that a cheap, bad red wine will have an abnormal extract, a high acidity, and no fine taste, and a good French claret will have a low acidity, a moderate extract, and a beautiful flavour. We have said nothing upon the subject of the genuineness of wine because we could not well discuss this subject without entering into the many ways in which wine may be adulterated. To enter fully into this subject would protract this article to undue length; but we may say that there is no evidence that the French clarets which we have examined are other than genuine wines. Indeed the wine production of the Gironde district is so enormous that there would be little object in adulteration.

### French White Wines.

*Sauternes and White Graves.*—These wines are grown on the right bank of the Garonne, chiefly on a gravelly soil—hence the name Graves. There are two main classes of Sauternes, the sweet Sauternes and the dry Sauternes. The sweet Sauternes owe their character to the fact that the grapes are not gathered until several weeks after they are ripe. This exposure of the grapes to the sun causes them to shrivel and to lose a good deal of water. The result is that they contain much more sugar than ordinary ripe grapes. At the same time a peculiar micro-organism developes on the grapes, which gives them the extraordinary bouquet characteristic of the sweet Sauternes. Formerly it was invariably the custom to make only this sweet type of Sauternes, but in more modern times, owing to the demand for a dry wine, dry Sauternes are also made. These retain very little sugar, but possess much of the characteristic flavour of the sweet Sauternes. The White Graves possess characteristics of their own owing to the soil, etc., but otherwise they are made in very much the same way as other dry white wines. These white Bordeaux wines possess a character quite distinct from the white wines of the Rhine or Moselle. They contain very little tannin, but otherwise do not differ very much in their general composition from the general run of the wines of the Bordeaux region, except perhaps that they are, on the whole, slightly more full bodied. Lighter wines are also made.

The analyses given above are good examples of sweet and dry Sauternes, and of two Graves wines—namely, the Haut Graves and the Graves.

### German Wines; Moselles and Hocks.

In the case of German wines this presumption certainly does not apply, and the amounts of certain brands of Hock and Moselle sold is largely in excess of their production in the very limited area concerned. Moreover, in Germany the addition of sugar and sugar solution to the must, and subsequent re-fermentation with great increase of the wine produced is officially recognised, and indeed legal. It would be invidious to dwell further upon this subject, but we may say here, instead of later on, that there is evidence that this process, so-called elongation, has been applied to some of the German wine we have examined. If we compare the French clarets with light German wines (Moselles and Hocks) we find that, so far as their alcohol content is concerned, there is little difference. As regards acid, however, the German wines contain about 50 per cent. more acid than the Bordeaux wines. In the case of some of the German wines this difference is more marked, and in addition some of the Moselles and Hocks contain an amount of sugar which cannot be regarded as negligible. Some of the latter have an exceedingly fine flavour, but the dictum which we expressed above holds true, that in this class of wine flavour is associated with a higher acid and a higher extract value than in the case of good claret.

### Champagne.

The next group of wine, of which we have analysed various samples, is champagne. A wine containing carbon dioxide under pressure was first produced on the commercial scale in the champagne district of France, hence the generic term champagne for effervescing wine. It is interesting in this connection to observe that the first person to produce a clear champagne by the process of "dégorgement" which is still used—that is by shaking the yeast residue out of the neck of the inverted bottle—was a father of the convent of St. Peter at about the end of the seventeenth century. Although champagne leaves much to be desired from the medical standpoint cf a beverage, it is a very favourite wine, and a short description of its manufacture will probably not be without interest to our readers.

(1) *The Choice of Grapes.*—This is very important, as some of the flavour and some of the tint of the finished product are due to the original grapes. The grapes most in demand for champagne are blue grapes, but grapes not particularly rich in colouring matter. It is very important that the grapes should be selected and inferior individuals rejected.

(2) *Fermentation and Addition of Sugar.*—The grapes are carefully pressed, care being taken not to press the skins and stones, and the resulting must allowed to ferment until only traces of unfermented sugar remain. The resulting young wine (cuvée), when cleared, forms the base wine of the champagne. To this a definite quantity of refined cane sugar is generally added; different quantities are added to different wines according to the amount of carbon dioxide they can absorb. The wine contains sufficient yeast to ferment this added sugar, but occasionally pure yeast culture is added. The next process the wine undergoes is fermentation, which takes place in the bottle and results in the absorption by the wine of the developed carbon dioxide. When the process is complete the yeast residue, which if allowed to remain would make the wine muddy, is first, by gradually changing the inclination of the bottles in the cellar, allowed to collect in the neck of the bottles. The bottles are then uncorked and the residue removed by various means. The last stage consists in what is known as dosing. This is essentially an addition of various quantities of liqueur, whereby the charmpagne gets its sugar and increase of its alcohol strength and an important part of its taste. The liqueur consists for the most part of a solution of candied sugar in wine and brandy, to which small portions of various other dessert wines are added. The cane sugar of the liqueur becomes, by keeping, partly or entirely inverted, and there seems to be no doubt that some of the taste of old champagne is due to this phenomenon. The pressure of the carbon dioxide in champagne varies in different brands, but it is always considerable, and amounts to from four to five atmospheres.

If we look at our own analyses of champagne we see that they all have a large amount of extract, and also a very considerable quantity of sugar, even the so-called extra dry wines. We should like to emphasise that merely chemically the taste of a champagne is by no means invariably a criterion of the amount of sugar it contains, but much more frequently of its acidity. For instance, in the extra dry champagne which we examined the sugar is very high, and also the total acidity. Such a champagne might taste quite dry on account of its acidity masking the whole of its sugar content. Invert sugar is much less sweet to the taste than cane sugar, and in some old originally sweet champagnes the sweet taste has become considerably modified by the inversion of their cane sugar. In drinking champagne one must always remember one is *ex hypothesi* disobeying a cardinal law of gastronomics, namely, the inadvisability of mixing one's alcoholic drinks. It is quite impossible to say how many varieties of wine one is taking at once when one drinks champagne, because the liqueur with which champagne is dosed is an exceedingly complex solution: quite apart from the fermentable sugar which it contains, the results of chemical analyses is really no criterion of its nature. We shall enter further into this subject as we come to consider the results of our physiological experiments.

### Australian, Spanish, and Italian Clarets.

The last class of wine to which we have directed our attention are the Australian, Spanish, and Italian clarets. We have purposely examined a large number of samples of Australian claret. These wines really must be considered in a class by themselves; the name claret or burgundy is quite inappropriate to describe them. They cannot be called light wines, for they contain nearly as much alcohol as port or sherry, a very high extract, and practically always a considerable quantity of sugar. With regard to the Spanish and Italian wines which we have analysed, we have not much to say, except that they, too, are in no sense comparable to French clarets. They are rich in extract and sugar and have no corresponding bouquet or flavour.

### A Physiological Investigation.

Finally, we have been concerned with experiments to determine the effect of different wines upon the chemical processes of digestion, and with a discussion of the results obtained. We will admit that this only covers part of the action of wines upon the human subject in that a question naturally of importance is not only how wines affect digestion, but also what is the remote effect of the constituents of wine after they have been absorbed from the stomach or the intestine. The effect of alcohol we have already considered, and what is known concerning the effect of extract, acid, and sugar we shall discuss in this connection. It has already been shown by many observers that alcohol in strengths up to approximately 5 per cent. has little or no effect upon the chemical processes of digestion. In fact, it may be regarded as stimulating rather than inhibitory. As we have investigated this matter rather more thoroughly than has previously been done, and as the technique in these experiments is identical with that adopted in the subsequent ones, we purpose giving our experiments.

In the experiments which follow, the starch used was Lintner's soluble starch and the diastatic ferment was Benger's liquor pancreaticus. For the peptic, pancreatic, and pepto-pancreatic experiments the proteid used was pure casein; the peptic solution was Benger's liquor pepticus and the pancreatic solution Benger's liquor pancreaticus. The time allowed for peptic digestions was one hour, for pancreatic three hours, for pepto-pancreatic one hour and subsequently three hours, for diastatic five minutes. The method adopted in the proteolytic digestions was to estimate the undigested residue quantitatively, and then the proteid digestion by subtraction. In the diastatic digestions the actual sugar formed was estimated in some cases by the rotatory index, and in others gravimetrically. Digestion was in each case stopped at the end of the time desired by bringing the material to the boil. In the case of pepto-pancreatic digestions, peptic digestion was concluded at the expiration of an hour, the undigested residue collected, neutralised, and then submitted for three hours to the pancreatic fluid. The same quantity of digesting fluid was used in all experiments, the results of which are compared; this was generally 5 c.c., the volume of the digestive mixture was generally 50 c.c. The quantity of the digesting agent was chosen after many preliminary experiments as being the one which, while well within physiological limits, best showed the differences in the cases of the different wines. The quantities of alcohol and wine chosen were those which were thought to correspond to the quantities of these substances likely to be present in the stomach or duodenum of a subject taking moderate quantities of wine or alcohol with his meals. This was a very difficult factor to estimate for two reasons: because alcohol is rapidly absorbed from the stomach, and hence the quantity of it present during a meal is constantly changing, and because the volume of the gastric contents is constantly increasing. Probably an average figure for the gastric contents during a meal is 1.5 litre, and an average dose of wine is, at the most, 10 ounces, so that, remembering that alcohol is gradually absorbed during digestion, the percentage of wine present in the stomach probably never at any moment exceeds 2 per cent. This is the figure we have taken for our experiments. We shall point out later on that we consider this question of dose most important, and that whether wine is good, bad, or indifferent to the consumer depends largely upon the dose. If we want to imitate, for experimental purposes, the quantity of alcohol present in the stomach in those subjects who take spirits with their meals, we must, of course, make quite a different calculation. An average peg of whisky is about two ounces, roughly 60 c.c. Its strength varies, but we can take the alcohol as 30 c.c. The consumption of this amount during a meal would, therefore, give us the same percentage in the gastric content as the consumption of 10 ounces of wine; in other words, the alcoholic percentage would not exceed 2 per cent. In comparing spirits with wine we must remember that in dealing with the former beverages we are dealing with a preparation of alcohol at least four, if not five, times as strong as the latter. It is true that spirits, except in the case of liqueurs, are generally drunk diluted. A "split" soda measures about six ounces, and a full bottle of soda-water or a half-bottle of Apollinaris about 10 ounces, so in the case of a whisky and soda we are generally dealing with a beverage of greater alcoholic strength than light wine. However, a small difference in the original dose of whisky makes a great difference in the alcoholic percentage in the stomach, and in dealing with it we are dealing with a beverage of sufficient alcoholic strength to demand accurate dosage, much more than with wine. As a matter of fact, in the ordinary household, the wine-glasses are of specific volume and correspond to what we may term the physiological unit of the respective wine, for instance, sherry, port, champagne, and claret glasses. We have no whisky-glasses, and are hence brought face to face with the absurdity that for the strongest form of alcohol which we use dietetically, we have not even a conventional measure.

### THE EFFECT OF ALCOHOL UPON ARTIFICIAL DIGESTION.

In these experiments we reckoned the amount digested of proteid or starch, as the case might be, as 100 when the digestive mixture was made with water. The quantity digested in the case of the mixtures containing wine is expressed as a percentage of this.

(*a*) *Diastatic digestion.*—Alcohol 2 per cent.: Sugar formed in unit time 104.6 per cent. Water: Sugar formed in unit time 100 per cent.

(*b*) *Peptic digestion.*—Three per cent. alcohol: Proteid digested in one hour 100.5 per cent. Water: Proteid digested in one hour 100 per cent.

(*c*) *Peptic and Pancreatic digestion.*—Two per cent. alcohol: Proteid digested 109.7 per cent. Water: Proteid digested 100 per cent.

From these experiments it is obvious that alcohol approximately up to 3 per cent. has a stimulating rather than an inhibitory influence upon the chemical processes of digestion.

TABLE II.—THE EFFECT OF CLARETS UPON PEPTIC DIGESTION.

| Wine and its Contents. | Per Cent. of Wine in Mixture. | Per Cent. of Proteid Digested. | Per Cent. Digested with Water. |
|---|---|---|---|
| (1) Médoc* ... ... | 20 | 85·5 | 100·00 |
| (2) Château d'Issan ... | 20 | 105·2 | 100·00 |
| (3) Ch. Brane-Cantenac | 20 | 97·8 | 100·00 |

* For the chemical composition of individual wines, see Table II.

TABLE III.—THE EFFECT OF CLARETS UPON PEPTIC AND PANCREATIC DIGESTION SUCCESSIVELY.

| Wine and its Contents. | Per Cent. in Mixture. | Per Cent. of Proteid Digestion. | Per Cent. Digested with Water. |
|---|---|---|---|
| (1) Ch. Larose ... ... | 15 | 96·6 | 100·0 |
| (2) Ch. Brane-Cantenac | 15 | 96·0 | 100·0 |

TABLE IV.—THE EFFECTS OF CLARETS UPON THE DIGESTION OF STARCH.

| Wine and its Contents. | Per Cent. of Wine in Mixture. | Per Cent. of Starch Digestion. | Per Cent. Digested with Water. |
|---|---|---|---|
| (1) Médoc ... ... | 10 | 103·6 | 100·0 |
| (2) Ch. d'Issan ... ... | 10 | 113·0 | 100·0 |

In the last two experiments it will be seen that we have taken a much smaller quantity of wine; we have done this because the digestion which these experiments imitates takes place in the duodenum, and supposing wine to be ingested with food, certainly half of it would be absorbed before the gastric contents left the stomach.

If we review shortly the result of our experiments so far as concerns the effect of claret upon the chemical processes of the digestion, we must come to the conclusion that on peptic digestion it has very little effect. The only distinct instance in which claret seemed to have an inhibitory effect upon peptic digestion was in the case of rather a crude claret possessing a high extract value and a high acidity; and the inhibitory effect of this particular sample upon digestion is probably dependent upon its tannic acid contents.

### French v. German Wines.

The next experiments deal with the effects of claret upon digestion as compared with those of hock and moselle. Before giving the details of these experiments we should like to emphasise the fact that the absolute amounts of the respective substances digested in the different batches of experiments with apparently the same quantities of the digested ferments are different, and this difference is probably due to inconstancy of the action of the ferment concerned. Results are compared, however, only when the same ferment in precisely the same strength has been used.

Table V.—Effect of Moselles, Hocks, and Clarets upon Peptic Digestion.

| Wine. | Strength. | Time of Digestion. | Amount Digested. | Amount Digested with Water. |
|---|---|---|---|---|
| | % | Hour. | | |
| Light Moselle ... | 20 | 1 | 115·00 | 100·0 |
| Schwarzburger ... | 20 | 1 | 106·00 | 100·0 |
| Moselle A ... ... | 20 | 1 | 127·00 | 100·0 |
| Hocheimer ... | 20 | 1 | 110·00 | 100·0 |
| Heavy Hock ... | 20 | 1 | 117·00 | 100·0 |
| Ch. d'Issan ... | 20 | 1 | 140·00 | 100·0 |

From the above table we see that in every case the amount of proteid digested when 20 per cent. of wine entered into the composition of the digestive mixture was greater than when this latter consisted only of water. We do not know that we attach very much importance to the actual numerical value of the results, but in view of statements so frequently made that wines have an inhibitory effect upon digestion, we think the above figures show clearly that this statement requires reconsideration. Further, the results we have obtained point clearly to the fact that a good claret is as beneficial and as stimulating to peptic digestion as a hock.

Table VI.—The Effect of Clarets, Hocks, and Moselles upon Peptic and Pancreatic Digestion.

| Wine. | Time of digestion. | Digested. | With water. | Remarks. |
|---|---|---|---|---|
| | Hours. | Per cent. | Per cent. | |
| 1. Ch. Larose | 1, peptic<br>3, pancreatic | 96·6 | 100·0 | A good light claret. 14 per cent. |
| 2. Ch. Brane-Cantenac | 1, peptic<br>3, pancreatic | 96·6 | 100·0 | A good light claret. 14 per cent. |
| 3. Hochheimer | 1, peptic<br>3, pancreatic | 98·0 | 100·0 | A good light hock. 14 per cent. |
| 4. Heavy Hock | — | 86·7 | 100·0 | A heavy hock. 14 per cent. |
| 5. Moselle, 1893 | 1, peptic<br>3, pancreatic | 98·9 | 100·0 | A good Moselle. 14 per cent. |
| 6. Moselle, A. | 1, peptic<br>3, pancreatic | 71·0 | 100·0 | A poor Moselle. 14 per cent. |

The results obtainable by means of artificial peptic digestions are in the case of wines obviously the most important, because they are most directly in accord with what actually happens when wine is being consumed at meal times; nevertheless, the residue of gastric digestion is, during life, submitted to pancreatic digestion, and although it is practically impossible to form anything but a rought estimate of how much of the wine consumed at a meal may reach the duodenum and then be present during pancreatic digestion, we thought it would be of interest in this, as in other cases, to compare the effects of the various wines upon the peptic and pancreatic digestion of the same proteid.

There is not a very clear inference to be drawn from the above experiments. We shall probably be correct in inferring that whatever influence is exerted by wine upon the pancreatic digestion of the gastric residue is due to the extract and acid moieties of the respective wine. If we compare the results obtained from the different wines we find an appreciable difference occurring only in two cases. The first influence was exerted in the case of a heavy hock (hock 4 of the Table). This, although a perfectly genuine hock, contained much extract and much acid. The greatest effect, however, seemed to be produced by Moselle A. This effect is somewhat difficult to explain chemically, and we think is probably due to the fact that the wine in question was not a genuine wine, containing an acidity high in proportion to its extract and strength. Before leaving this part of the subject we would note that good claret seems practically without influence upon the pancreatic digestion of the gastric residue.

### Amylolytic Digestion.

We made several experiments upon the effects of hock, claret, and moselles upon the conversion of starch into sugar by the pancreatic juice. Here we obtained results of some interest; good red wine and full-bodied hock in quantities up to about 6 per cent. stimulated the conversion of starch into sugar. Above this strength the clarets have only a slightly less effect than in the former case, but the heavy hocks, and indeed moselles, begin to exert a marked inhibitory influence. This will be apparent in the following table:—

Table VII.—The Influence of Claret, Hock, and Moselle upon the Pancreatic Digestion of Starch.

| Wine | Strength. | Time. | Quantity of Sugar Produced. | Quantity Produced by Control. |
|---|---|---|---|---|
| | % | Hour. | | |
| Château Larose ... | 4 | ½ | 129·9 | 100·00 |
| | 6 | ½ | 131·5 | 100·00 |
| | 8 | ½ | 126.5 | 100·00 |
| Heavy Hock, No. 4 | 4 | ½ | 129·9 | 100·00 |
| | 8 | ½ | 56·4 | 100·00 |
| Moselle, '93 ... | 6 | ½ | 119·0 | 100·00 |
| | 8 | ½ | 86·9 | 100·00 |

The next series of experiments were designed to compare the effect of champagne and claret upon the different digestions. Champagne is a very favourite wine, and its varieties are legion, so that there is no lack of material for experiments. After a number of preliminary analyses we chose four champagnes, which appear in our general analytical table as champagnes 1, 2, 3, and 4. The first three of these wines were fairly high priced, and were average in quality both with regard to their chemical composition and their taste. The last (champagne 4) was a cheap full-bodied champagne, containing a considerable percentage of sugar and also a hypernormal quantity of acid.

Table VIII.—Influence of Claret and Champagne upon Peptic Digestion.

| Wine. | Strength. | Time. | Amount Digested. | Amount Digested in Control. |
|---|---|---|---|---|
| | % | Hour. | | |
| Ch. d'Issan ... | 15 | 1 | 105·0 | 100·00 |
| Champagne 1 ... | 15 | 1 | 115·0 | 100·00 |
| ,, 2 ... | 15 | 1 | 130·0 | 100·00 |
| ,, 3 ... | 15 | 1 | 106·0 | 100·00 |
| ,, 4 ... | 15 | 1 | 120·0 | 100·00 |

Before discussing the results obtained in the experiments corresponding to Table VIII. we will proceed to give a table showing the effects of claret and champagne upon peptic and pancreatic digestions consecutively.

Table IX.—The Influence of Clarets and Champagnes upon Peptic and Pancreatic Digestions.

| Wine. | Strength. | Time of Digestion. | Amount Digested. | Amount Digested by Control. |
|---|---|---|---|---|
| | % | Hours. | | |
| Ch. Larose ... | 17 | 1 & 3 | 96·6 | 100·00 |
| Ch. Brane-Cantenac | 17 | 1 & 3 | 96·6 | 100·00 |
| Champagne 1 ... | 17 | 1 & 3 | 68·6 | 100·00 |
| ,, 2 ... | 17 | 1 & 3 | 68·6 | 100·00 |
| ,, 3 ... | 17 | 1 & 3 | 65·4 | 100·00 |
| ,, 4 ... | 17 | 1 & 3 | 78·4 | 100·00 |

Upon reference to the above tables two inferences seem justifiable. These are that champagne in moderate doses is at any rate capable of stimulating the digestion of proteids in the stomach. This influence seems to be greater than that of clarets or hocks, although if we turn back for a moment to Table 5 we shall find that the figures there, using 20 per cent. of wine, closely approximate to, and in some cases even exceed, those obtained with champagne. It is hardly accurate to compare one table with another, and we think that the inference that champagne under certain circumstances does stimulate proteid digestion in the stomach to a greater degree than still wines is justified. This is in part at least dependent upon the acid, but probably the carbon dioxide has a mechanical stimulating effect even *in vitro* and exerts this effect to a greater degree *in vivo*.

### Pancreatic Digestion.

If we turn, however, to the result of the pancreatic digestion of the gastric residue, a second inference from our experiments seems justified—namely, that champagne exerts an inhibitory influence upon digestion in the duodenum. The results we obtained in this direction are, we think, too definite to be within the limits of experimental error. It appears that if we assume, as we must assume for the sake of these experiments, that the extract moiety of wines taken at meal-times reaches the duodenum, and is present there during the whole time of the pancreatic digestion of the gastric residue, it always exerts a slightly unfavourable influence. However, in the case of clarets, light hocks, and moselles this influence is so small as to be negligible, but in the case of champagne and heavy hocks it may be considerable. Any influence interfering with duodenal digestion is a matter of considerable importance, because the by-products of the tryptic digestion of proteids are in many cases active substances, and the malaise occasionally following the consumption of heavy wines and champagne may be due to this identical cause.

### Californian and Other Wines.

The last branch of the subject, as far as wines are concerned, deals with the comparison between French clarets and Australian, Californian, Spanish, and Italian red wines. When we discussed the chemical composition of these latter beverages we pointed out that the term "claret" was hardly applicable to them, in that their alcohol was greatly in excess—sometimes, indeed, almost double—of that normally present in French claret. Their extract and acid values are also high, and most of them contain considerable percentages of sugar.

Table X.—The Effect of French, Australian, and Californian "Clarets" upon Peptic Digestion.

| Wine. | Strength. | Time. | Amount Digested. | Amount Digested by Control. |
|---|---|---|---|---|
| | % | Hour. | | |
| Ch. d'Issan ... | 20 | 1 | 105·00 | 100·00 |
| Californian ... | 20 | 1 | 85·4 | 100·00 |
| Australian, 1 ... | 20 | 1 | 69·5 | 100·00 |
| ,, 2 ... | 20 | 1 | 98·3 | 100·00 |
| ,, 3 ... | 20 | 1 | 83·9 | 100·00 |
| ,, 4 ... | 20 | 1 | 88·6 | 100·00 |
| ,, 5 ... | 20 | 1 | 81·9 | 100·00 |
| ,, 6 ... | 20 | 1 | 97·00 | 100·00 |

Table XI.—Effect of French, Australian, Californian, Spanish, and Italian Clarets upon Peptic and Pancreatic Digestion.

| Wine. | Strength. | Time. | Amount Digested. | Amount Digested by Control. |
|---|---|---|---|---|
| | % | Hour. | | |
| Ch. Larose ... | 15 | 1 | 96·5 | 100·00 |
| Californian ... | 15 | 1 | 79·2 | 100·00 |
| Australian, 1 ... | 15 | 1 | 71·3 | 100·00 |
| ,, 2 ... | 15 | 1 | 59·5 | 100·00 |
| ,, 3 ... | 15 | 1 | 56·1 | 100·00 |
| ,, 4 ... | 15 | 1 | 57·0 | 100·00 |
| ,, 5 ... | 15 | 1 | 63·2 | 100·00 |
| ,, 6 ... | 15 | 1 | 55·3 | 100·00 |
| ,, 7 ... | 15 | 1 | 56·2 | 100·00 |
| Spanish ... ... | 15 | 1 | 73·7 | 100·00 |
| Italian ... ... | 15 | 1 | 72·2 | 100·00 |

By reviewing the above two tables we see that our former results are essentially confirmed, and that the clarets, other than the true French claret, probably by virtue of their high acidity and sugar content exert a slight, but distinct, inhibitory influence upon the digestion of proteids. This influence is far more marked in pancreatic digestion than in peptic, and, in the living subject, would be more or less marked according to the extent to which the extract was absorbed in the stomach. As we saw above, a similar influence upon pancreatic digestion was exerted by champagne, but the stimulating effect of this latter wine upon peptic digestion is entirely absent in the case of the non-French clarets. The unfavourable influence which the above wines exert upon peptic digestion is, we think, due to the quality, and not the quantity, of their extract, and probably to its tannin derivatives.

### Tea and Ginger Beer: Wines compared with some Other Beverages.

The last part of our subject concerns itself with a comparison between the effect of wines (claret) and tea and ginger-ale upon the processes of digestion. Both the latter beverages are very frequently taken at meal times. The consumption of tea in this country has during the last ten years increased enormously. As the nutritive value of this drink, if we eliminate the sugar and milk taken with it, is practically *nil*, we must regard the alkaloid which it contains in every sense as a stimulant, or beverage capable of unlocking, but not of supplying, energy. The leaves from which tea is made vary considerably in composition according to their age and the time of year at which they are gathered. Further, tea as we drink it also varies in composition according to the time during which it is allowed to infuse. The most important constituents in tea are first of all the alkaloid (thein, 1:3:7-trimethylxanthin), and secondly tannin. The analyses of 158 samples of tea-leaves showed that the quantity of thein in them varied between 1.09 to 4.67 per cent., the average content being 2.79 per cent. The tannin in the same samples varied from 4.48 to 25.20 per cent., the average being 12.35.

### A Cup of Tea.

In an ordinary cup of tea, reckoning the volume of this at six ounces, one could easily get 2½ grains of thein and 20 grains of tannin (König, vol. i. page 1016); that is, approximately a full Pharmacopœial dose of both of these drugs. There is very good reason to suppose that, although the injurious effects of tea are not so apparent as those of alcohol, they nevertheless exist. Recent statistics show that the consumption of tea is greatest in Australia, where it reaches about eight pounds per head of the population per annum. As we shall point out later on, tannin and its derivatives are substances which exert a particularly unfavourable influence upon digestion, in that they not only form insoluble compounds with the proteid elements of food, but also actually precipitate the enzymes which render food soluble and capable of being absorbed. The exact action of tea upon the digestive ferments has, so far as we know, not been accurately worked out, and such a research would, in our opinion, have results of considerable importance to society at large.

### Ginger Beer.

The temperance beverage which we have chosen for the purpose of this research is ginger-ale. The ginger in this beverage would probably have an effect in the living subject which would not be shown in our experiments in glass.

Table XII.—Influence of Claret, Tea, Ginger-beer, and Ginger-ale upon Peptic Digestion.

| Beverages. | Strength. | Time. | Amount Digested. | Amount Digested by Control. |
|---|---|---|---|---|
| | % | Hour. | | |
| Claret (Ch. d'Issan) | 20 | 1 | 105·2 | 100·00 |
| Tea ... ... | 20 | 1 | 68·1 | 100·00 |
| Ginger-beer ... | 20 | 1 | 97·4 | 100·00 |
| Ginger-ale ... | 20 | 1 | 98·4 | 100·00 |

Table XIII.—Effect of Claret, Tea, Ginger-beer, and Ginger-ale upon Peptic and Pancreatic Digestion.

| Beverages. | Strength. | Time. | Amount Digested. | Amount Digested by Control. |
|---|---|---|---|---|
| | % | Hours. | | |
| Claret (Ch. Larose) | 15 | 1 Peptic. 3 Panc. | 96·6 | 100·00 |
| Ch. Brane-Cantenac | 15 | do. | 96·0 | 100·00 |
| Tea ... ... | 15 | do. | 90·0 | 100·00 |
| Ginger-beer ... | 15 | do. | 84·0 | 100·00 |
| Ginger-ale ... | 15 | do. | 80·0 | 100·0C |

Table XIV.—Effect of Claret and Tea upon the Pancreatic Digestion of Starch.

| Beverages. | Strength. | Time. | Amount Digested. | Amount Digested by Control. |
|---|---|---|---|---|
| | % | Hour. | | |
| Claret (Médoc) ... | 10 | ½ | 103·6 | 100·0 |
| Ch. d'Issan ... | 10 | ½ | 113·0 | 100·0 |
| Tea ... ... | 10 | ½ | 101·2 | 100·0 |

### The Results.

The results of the above experiments show what was *à priori* to be expected—namely, that tea has a distinctly unfavourable effect upon the digestion of proteids in the stomach, and, indeed, upon the digestion of the proteids by the pancreatic juice. Its effect upon the digestion of starchy foods is not nearly so marked, and it cannot be regarded as in any way hindering this. Ginger-beer and ginger-ale appear to inhibit the proteid digestion in the duodenum, while they have but little effect upon proteid digestion in the stomach. If we compare a light red wine with these beverages we see once again that, provided it is taken in small quantities, it is to be preferred upon physiological grounds.

The practice of taking tea with bread-and-butter and sweet things seems, from these experiments, to be justified in that it is precisely upon the class of digestion involved in these foods that tea has no unfavourable influence.

### GENERAL CONCLUSIONS.

### The Scope of the Inquiry and some Practical Results.

Those readers who have carefully followed the report will now be rightly getting anxious to hear the conclusion of the whole matter. When we began to write the article now almost terminated our intention was just to give in the text actual experimental results, and consider the inferences to be drawn from these *en bloc* at the end of the paper. As the research continued, however, it soon became apparent that so simple a procedure was impossible, and that the conclusions must be dealt with, and, so to speak, formulated both under their respective tables, and also at the end of the article.

In formulating these conclusions we feel that a heavy responsibility rests upon us, in that as medical men we must also consider that most variable, but yet clinically important, factor—namely, the taste of our patients. We have tried to do this and to adopt the maxim of Horace in the "Ars Poetica"—namely, to gain every vote by mingling the useful with the sweet." (*Omne tulit punctum qui miscuit utile dulci.*)

### Light Wines.

Our first conclusion, which, indeed, in many ways is the most important one, relates to all the wines we have examined with the exception of the Australian and Californian clarets, which are not really light wines (*vide infra*). It is that wine drinking with meals in doses of, roughly, 8 to 10 oz. (say, rather, more than a quarter, rather less than a half, bottle) during dinner or lunch is in no sense inimical to digestion. Wine should be sipped, and not drunk by draughts, and it is well to dilute it with approximately an equal quantity of ordinary good water, or alkaline mineral water, which should not be too strongly effervescent nor too cold. This dilution can take place just as well in the stomach as in the glass; in other words, there is no necessity to mix the diluent with the wine in the glass of the consumer. This detail is of a little importance gastronomically, in that alkaline waters and also tap water may, and often do, spoil the bright colour of either red or white wine.

### The Purity of Light Wines.

With regard to the genuineness of wine, we should be sorry to make sweeping assertions from insufficient analytical data, but our research has certainly led us to confirm the view expressed by other contemporary scientific papers—that the purchaser of French wines is extremely unlikely to get anything but a genuine wine. Our own chemical results and those of others, as well as considerations relating to the laws of production and demand, render adulteration *a priori* not improbable in the case of other light wines.

### A Practical Rule.

A conclusion from the digestive experiments which seems clear is that in the case of an overdose of wine it is to the acid and the extract that we must ascribe the inhibitory effects which may accrue; further, these effects are the more marked according as these constituents reach the duodenum in more or less quantity. The practical rule to be inferred from this is that patients suffering from weak gastric digestion or hyperacidity, must avoid highly acid wines and wines rich in extract. To recommend such a patient a given wine would be difficult; and we would in this respect tell him to drink, not the best wine, but the best wine he can digest, just as Solon made for the Athenians, not the best laws, but the best laws they could keep.

### Champagne.

Champagne stands in a special position. There can be no doubt that the good brands of champagne are powerful stimulants to gastric digestion, and hence are exceedingly useful to patients suffering from atonic dyspepsia. In these cases, however, the dose should be carefully regulated, and the wine should be sipped, that it may become thoroughly mixed with the gastric contents.

To patients suffering from amylaceous indigestion or any impairment of pancreatic function champagne should be given very cautiously, and its results carefully watched. To what extent its untoward effect upon pancreatic digestion may, however, be neutralised by the adequate administration of a suitable alkaline water may rightly be tried on any patient. We regard its unpleasant after effects as most probably due to its interference with duodenal digestion.

### Californian and Australian Wines.

The Californian and Australian clarets cannot be called light wines; their dose should be regulated according to their alcoholic content. They are no doubt useful as tonic beverages, but must be considered on the basis of port, sherry, or madeira, and used as such.

### Tea and Ginger Beer.

Our conclusions with regard to tea are that its original popular use as a stimulating beverage with breakfast and tea is justified and harmless, but that its indiscriminate consumption with meat foods, or during proteid digestion, is likely to be harmful. China tea, on account of its less tannic acid content, is less harmful than Indian tea. Our results with ginger beer showed that this beverage was practically without influence upon digestion. The experiments *in vitro*, however, probably placed it at a disadvantage, because the valuable carminative action of the ginger and its allies do not from the nature of the case come into play.

